# Regulation of Formation, Stemness and Therapeutic Resistance of Cancer Stem Cells

**DOI:** 10.3389/fcell.2021.641498

**Published:** 2021-04-07

**Authors:** Nan Jing, Wei-Qiang Gao, Yu-Xiang Fang

**Affiliations:** ^1^State Key Laboratory of Oncogenes and Related Genes, Renji-Med X Clinical Stem Cell Research Center, Renji Hospital, School of Medicine, Shanghai Jiao Tong University, Shanghai, China; ^2^School of Biomedical Engineering and Med-X Research Institute, Shanghai Jiao Tong University, Shanghai, China

**Keywords:** cancer stem cells, therapeutic resistance, tumor microenvironment, stemness, stemness maintaining

## Abstract

Over the past 20 years cancer stem cells (CSCs) have been proposed as key players in the tumorigenesis and progression, which are closely related to the initiation, metastasis and therapeutic resistance of cancer. Evidences have been provided that both genetic and epigenetic factors contribute to the regulation of the formation and stemness maintenance as well as the therapeutic resistance of CSCs via affecting various signal pathways. In addition, the interaction between CSCs and tumor microenvironment has also been revealed to be involved in the above-described processes. With the aim of targeting CSCs to improve treatment outcome, we herein discuss the mechanisms that orchestrate the characteristic of CSCs by the three elements and potential therapeutic strategies. We also summarize how several key regulatory factors function in the regulation of not only the formation and stemness maintenance, but also the therapeutic resistance of CSCs. Thus, future studies focusing on these key factors would be helpful for the development of novel drugs targeting CSCs.

## Introduction

Cancer stem cells hypothesis endorses that CSCs are a subset of cancer cell subpopulations in the tumor, which are considered to be responsible for tumor initiation, recurrence, metastasis, and therapeutic resistance (Visvader and Lindeman, 2012). Because of the key contribution of CSCs to tumor heterogeneity and consequential resistance to therapy (Kesh et al., 2020), it is urgent to explore underlying mechanisms. While mutations of driver genes are key to the initiation of CSCs, epigenetic factors such as DNA methylation (Toh et al., 2017) and non-coding RNAs deregulation (Wang Y. et al., 2015) on multiple stem cell signal pathways including the Wnt signaling (HOXA5 Counteracts Stem Cell Traits by Inhibiting Wnt Signaling in Colorectal Cancer), Notch signaling (Xiao et al., 2017), and Hedgehog signaling (Zhu and Wang, 2020) are of equal importance. In addition, a cell-to-cell communication between CSCs and other types of adjacent cells such as endothelial cells (Krishnamurthy et al., 2010), macrophages (Fang et al., 2017), and fibroblasts (Fiori et al., 2019) in the tumor microenvironment also play influential roles. In this review, we summarize genetic and epigenetic regulatory factors and tumor microenvironment inducers on the regulation of the formation, stemness maintenance and therapeutic resistance of CSCs, and propose potential strategies for cancer therapy targeting CSCs.

## Regulation of Formation of CSC

**FIGURE 1:**
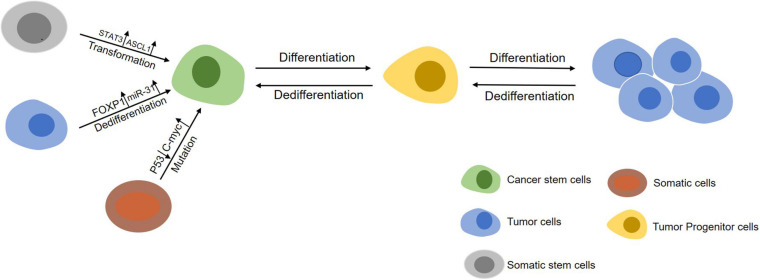
An overview of the initiation of CSCs. Somatic stem cells (gray) can maintain self-renewal through symmetrical divisions. However, normal stem cells may transform into CSCs (green) under following circumstances including gene mutations occuring in cells during chromatin rearrangements and influence by factors in the tumor microenvironment. On the other hand, differentiated cancer cells (blue) can dedifferentiate into a cancer stem-like cells state by abnormal signal activation or induction from other cells in tumor microenvironment.

Over the past two decades, regulation of CSCs formation by genetic, epigenetic and tumor microenvironmental elements has been highlighted (Figure 1). First, chromatin remodeling for gene rearrangement (Liau et al., 2017) and accumulation of gene mutations (Davies et al., 2011) by key compounds are major genetic transformations in somatic stem cells from normal tissues or bone marrow to regain phenotypes and profiling characteristics of CSCs. Second, epigenetic modification through methylation and non-coding RNAs can give rise to deregulation and gain-of-function in somatic stem cells to form a CSCs-like state. Third, soluble cytokines/chemokines secreted by adjacent cells (e.g., fibroblasts, endothelial cells, and immunocytes) in tumor microenvironment can also induce the initiation of CSCs (Table 1).

**TABLE 1:** Genes involved in the initiation of CSCs.

**Regulation model**	**Gene**	**Cancer type**	**Function**	**References**
Chromatin remodeling	*Ascl1*	Glioblastoma	Cell proliferation	Park et al., 2017
	*Morc2*	Gastric carcinoma	Changing the structure of chromosomes; DNA damage repair	Li et al., 2012; Wang Y. et al., 2015
Gene mutation	*P53*	Hepatocellular carcinoma	Tumorigenesis	Liu et al., 2017
	*c-myc*	Hepatocellular carcinoma	Tumorigenesis	Liu et al., 2017
	*Hoxc8*	breast cancer	Promoting cancer cell differentiation	Shah et al., 2017
	*Crebbp*	Lymphoma	Inhibiting the initiation of CSCs	Horton et al., 2017
	*Par3*	Prostate cancer	Regulating cell division pattern	Zhou et al., 2019
	*Zeb1*	Prostate cancer	Promoting EMT	Wang X. et al., 2020
	*Nf-kB*	Intestinal cancer	Inducing the dedifferentiation of non-stem cells	Schwitalla et al., 2013
	*Stat3*	Breast cancer	Tumorigenesis	Kim et al., 2019
Histone and mRNA methylation	*Kdm3*	Colorectal cancer	Promoting H3K4 methylation	Li et al., 2017
	*Suv39h1*	Lung cancer	Increasing H3K9 dimethylation	Wang Z. S. et al., 2020
	*Socs3*	Lung cancer	Enhancing transformation of CSCs and tumorigenesis	Wang Z. S. et al., 2020
	*Alkbh5*	Glioblastoma	Promoting CSCs proliferation	Zhang S. C. et al., 2017
Post-transcriptional regulation by microRNA	*miR-31*	Breast cancer	Promoting mammary epithelial cell proliferation	Lv et al., 2017
	*miR-200c*	Breast cancer	Promoting transformation of MaSC to breast cancer stem cells	Shimono et al., 2009
Post-transcriptional regulation by lncRNA	*Meg3*	Glioblastoma	Inhibiting cell growth and migration	Buccarelli et al., 2020
Induction by endothelial cells	*Nanogp8*	Colorectal cancer	Initiating CSCs	Wang et al., 2017
	*Ptk7*	Head and neck squamous cell carcinoma	Promoting the phenotype of CSC	Yu et al., 2018
	*Ar*	Prostate cancer	Promoting the transformation of CSCs	Liao et al., 2017

### Chromatin Remodeling

A key study has given evidences that in glioblastoma stem cells (GSCs) elevated expression of Achaete-Scute Complex-Like 1 (ASCL1) can promote the proliferation of quiescent cells and their differentiation into neurons by binding the chromatin at enhancer region of the neural target genes to activate the transcription of these target genes, including NK6 homeobox 2 (NKX6-2), high mobility group AT-hook 2 (HMGA2), G protein-coupled receptor 37-like 1 (GPR37l1) and myelin transcription factor 1 (MYT1). Among them, MYT1 can directly inhibit NOTCH signal during neural development of mice, indicating a coordinated role of ASCL1 in cell fate determination program (Park et al., 2017). Another study has reported that the MORC family CW-type zinc finger 2(MORC2) protein is a new chromosome remodeling protein that changes the structure of chromosomes in an ATPase-dependent manner to effectively repair DNA damage induced by ion irradiation (Li et al., 2012). Importantly, p21 (RAC1) activated kinase 1 (PAK1) protein kinase-mediated phosphorylation of MORC2 inhibits expression of tumor suppressor gene *p21* and cytoskeleton-related gene *Argbp2* to promote gastric tumorigenesis (Wang G. L. et al., 2015).

### Gene Mutation

As a key tumor-suppressive transcriptional factor, although the inactivation of P53 alone is not sufficient to drive hepatocellular carcinoma (HCC) tumorigenesis, evidences has been provided that inactivation of P53 along with overexpression of oncogenes such as c-Myc makes hepatocytes more prone to oncogenic transformation and to acquire CSCs characteristics so to increase stem genes expression (Liu et al., 2017). Another oncogenic driver gene homeobox C8 (Hoxc8) has also been reported as an important regulator of the formation of stem cells and can act as a regulator of breast cancer cells differentiation. Hoxc8 silencing endows mammary gland cells with stem cells potential and an increased CSCs population (Shah et al., 2017). On the other hand, cyclic AMP response element binding protein (CREBBP) was recently shown to be a novel inhibitor for the initiation of CSCs. It has been found that mice with early deletion of CREBBP in the hematopoietic stem and progenitor cells compartment shows changes in DNA damage response and increased frequency of invasive lymphoproliferative disorders/lymphoma. Loss of CREBBP leads to the subsequent development of mature lymphoid malignancies and the generation of cancer progenitor cells (Horton et al., 2017). Our previous work demonstrated that loss of pulmonary adenoma resistance 3 (Par3), a key cell polarity molecule, is tightly involved in the promotion of prostatic tumorigenesis, which is related to the change of cell division modes in cancer progenitor cells (Zhou et al., 2019). In addition to loss-of-function gene mutation, our recent studies also indicate that elevated expression of zinc finger E-box binding homeobox 1 (ZEB1), an important epithelial-to-mesenchymal transition related transcription factor, in prostate basal stem cells which is associated with the formation of CSCs in the prostate promotes androgen independence of prostate cancer (Wang X. et al., 2020). Similarly, upregulation of NF-kB was found to enhance the activity of the Wnt pathway and in turn to induce the dedifferentiation of non-stem cells and acquisition of cancer stem cells-like properties (Schwitalla et al., 2013). In addition, a recent study reported two missense mutations of CHD4 gene (CHD4^R975H^ and CHD4^R1162W^) that were located in the ATPase binding domain of CHD4. It was found that both mutations can reduce the half-life of CHD4 protein and inactivate CHD4 protein to promote the phenotype of CSCs through the TGF-β/CD133 pathway, expand the CSCs population, and enhance the progression of endometrial cancer (Li et al., 2018).

### Upregulation of Stemness Related Factors

A recent study showed that JAK2 can interact with PAK1 to regulate the nuclear translocation of PAK1 and Stat3. The Stat3/PAK1 complex is recruited to the IL-6 promoter and induces the transcription of IL-6 gene, a stemness related factor for the formation of BCSCs through JAK2/PAK1/Stat3/IL-6 signaling (Kim et al., 2019). Additionally, a recent study demonstrated that FOXP1 can function as an oncogene and a CSCs driver gene in epithelial ovarian cancer cells. FOXP1 upregulates the transcriptional activities of four key stemness factors, ATP Binding Cassette Subfamily G Member2 (ABCG2), octamer-binding transcription factor 4 (OCT4), nanog homeobox (NANOG) and SOX2, to promote CSCs-like features in ovarian cancer cells (Choi et al., 2016).

### Histone and mRNA Methylation

It has been well reported that the change of post-translational modification profiling, especially the methylation of histones is tightly associated with tumorigenesis. Recently, loss of histone methylation in specific gene promoters has been indicated to be associated with the formation of CSCs and. For example, the histone demethylases of the lysine demethylase 3 (KDM3) family, including KDM3A, KDM3B and JMJD1C, can remove methyl groups from H3K9me2 and simultaneously recruit histone methyltransferase MLL1 to promote H3K4 methylation and then enhance the transcription of Wnt target genes. KDM3 family has also been found to play a key role in the carcinogenic potential of CSCs by regulating Wnt/β-catenin-mediated transcription. In contrast, depletion of KDM3 significantly inhibits the germinal potential and survival of CSCs (Li et al., 2017). In addition, studies have shown that co-exposure of arsenic and benzo-a-pyrene has a synergistic effect on the induction of malignant transformation of CSCs in tumorigenesis by increasing expression of histone H3 lysine 9 methyltransferase (SUV39H1). Upregulation of SUV39H1 increases H3K9 dimethylation (H3K9me2) to decrease expression of tumor-suppressive suppressor of cytokine signaling 3 (SOCS3), which leads to a significantly enhanced activation of Akt and Erk1/2 for transformation of CSCs and tumorigenesis (Wang Z. S. et al., 2020). On the other hand, N6 methyltransferase and demethylase can regulate gene expression and cell fate through dynamic and reversible N6 methyladenosine (m6A) RNA modification. Evidences have been found that the m6A demethylase alkB homolog 5 (ALKBH5) is highly expressed in CSCs. Silencing ALKBH5 can inhibit the proliferation of CSCs. ALKBH5 enhances its expression by demethylating the nascent transcripts of forkhead box M1 (FOXM1) and the relevant non-coding RNA, FOXM1-AS. FOXM1-AS can promote the combination of ALKBH5 and FOXM1 nascent transcripts. Deleting ALKBH5 and FOXM1-AS blocks the tumorigenicity of CSCs (Zhang S. C. et al., 2017).

### MicroRNA

Accumulating researches indicated that dysregulation microRNA is an early event involved in the initiation of CSCs and tumorigenicity (Nasr et al., 2019; Yan D. J. et al., 2019; Yan X. L. et al., 2019). Recently, miR-31 has been found to be highly expressed in mammary stem cells (MaSCs) and breast cancer. It promotes mammary epithelial cell proliferation and MaSCs expansion *in vivo* by regulating various signaling pathways such as TGF-β and Prlr/Stat5. While defected miR31 affects the growth of breast cancer, reduction of CSC numbers and metastasis of the tumors to the lung (Lv et al., 2017), miR-200c has been indicated to be involved in the self-renewal process of stem cells by regulating expression of BMI and Suz12 genes. In addition, miR-200c has been identified as an important trigger for the transformation of MaSCs to breast cancer stem cells (BCSCs). Restoration of its expression can inhibit the clonality of BCSCs *in vitro* and the tumorigenicity *in vivo* (Shimono et al., 2009).

### LncRNA

Similar to microRNA, lncRNA is also one of the key regulators of CSCs (Castro-Oropeza et al., 2018). In glioblastoma (GBM), lncRNA MEG3 acts as a tumor suppressor and its low expression is significantly related to the short survival of GBM patients. Restoring expression of maternally expressed 3 (MEG3) inhibits cell growth and migration, thereby reducing the tumorigenic effect of GSCs and their invasive growth (Buccarelli et al., 2020).

### Endothelial Cells

It has been reported that liver parenchymal endothelial cells can mediate the initiation of CSCs in colorectal cancer (CRC) in a paracrine manner by activating the Nanog homeobox retrogene P8 (NANOGP8) pathway (Wang et al., 2017). Additionally, conditioned medium from endothelial cells derived from tumor microvessels can restore the CSC phenotype of differentiated GBM, which is mediated by bFGF, a major soluble factor present in the conditioned medium (Fessler et al., 2015).

### Macrophages

In tumor microenvironment, it has been found that IL-33 induces macrophages to infiltrate into tumor tissues to produce prostaglandin E2 which makes tumor cells to acquire CSCs’ stemness and to support proliferation of CSCs in CRC (Fang et al., 2017).

### Cancer-Associated Fibroblasts (CAFs)

It has been shown that CAFs can increase the number of liver CSCs through a paracrine manner in which hepatocyte growth factor (HGF) activates c-Met/FRA1/HEY1 signaling to develop a fibrosis-dependent HCC (Lau et al., 2016). In head and neck squamous cell carcinoma (HNSCC), it was recently found that CAFs secretes periostin that significantly upregulates the CSCs-like phenotype, proliferation and invasion in HNSCC. Mechanically, periostin binds to protein tyrosine kinase 7 (PTK7) on the cell membrane of CSCs. This interaction activates downstream Wnt/β-Catenin signaling to promote the phenotype of CSCs (Yu et al., 2018). In prostate cancer, androgen receptor can also be activated by prostate CAFs-secreted interferon-γ (IFN-γ) and macrophage colony stimulating factor (M-CSF), which promotes the expression of stem cell markers in prostate cancer cells to acquire the characteristics of prostate cancer stem-like cells (PCSCs) (Liao et al., 2017).

## Regulation of Stemness of CSC

**FIGURE 2:**
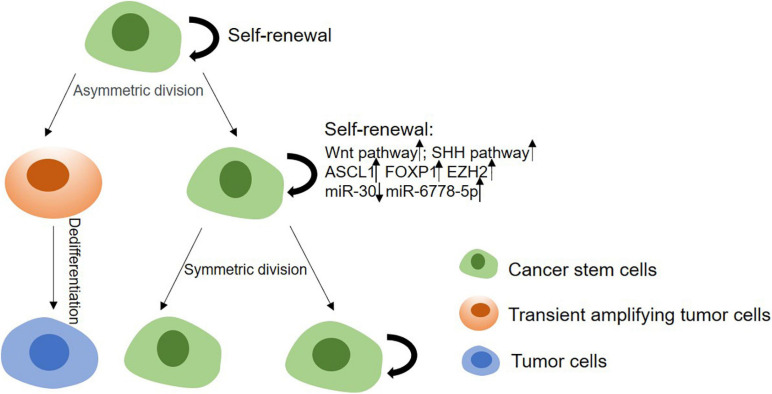
The mechanism of stemness maintenance of CSCs. In CSCs model, CSCs can progress into solid tumors due to their self-renewal and proliferation capabilities. CSCs can give rise to transient amplifying cells, and then develop into committed pre-malignant progenitors, differentiated malignant cells. On the other hand, CSCs can renew and proliferate themselves through upregulating Wnt, Shh, Notch pathways, or enhancing DNA damage repair ability, etc.

Cancer stem cells’ stemness is responsible for tumor initiation capability, metastasis and therapeutic resistance. Thus, it is important to study the mechanism of regulating the stemness of CSCs for a better therapy of tumors (Figure 2). Enhancement of CSCs’ stemness can be caused by activated stemness related signaling pathways, enhanced the capability of DNA damage repair or disordered epigenetic regulation of methylation and non-coding RNAs, and also can be induced by tumor microenvironment (Table 2).

**TABLE 2:** Genes involved in the stemness maintenance of CSCs.

**Regulation model**	**Gene**	**Cancer type**	**Function**	**References**
Gene mutation	*Trib3*	Colorectal cancer	Changing the homeostasis of CSCs’ self-renewal and differentiation	Hua et al., 2019
	*Phf20*	Glioblastoma	Sustaining the stem cell-like phenotypes	Ma et al., 2020
	*Shh*	Pancreatic cancer	Sustaining the characteristics of CSCs	Miyazaki et al., 2016
DNA damage repair	*Rad6*	Ovarian cancer	Regulating mutagenic DNA damage tolerance	Somasagara et al., 2017
DNA and histone methylation	*Prmt5*	Breast cancer	Enhancing the stemness of CSCs	Chiang et al., 2017
	*Foxp1*	Breast cancer	Enhancing the stemness of CSCs	Chiang et al., 2017
	*Set1*	Breast cancer	Enhancing the stemness of CSCs	Chiang et al., 2017
	*Ezh2*	Glioblastoma Breast cancer	Promoting the self-renewal and tumorigenetic ability of CSCs	Suva et al., 2009; van Vlerken et al., 2013
Post-transcriptional regulation by microRNA	*miR-30*	Breast cancer	Inhibiting the self-renewal of CSCs	Yu et al., 2010
	*miR-7*	Prostate cancer	Promoting tumor growth and metastasis	Chang et al., 2015
	*miR-6778-5p*	Gastric cancer	Mediating the compensatory activation of cytoplasmic carbon metabolism	Zhao et al., 2020
Formation RNA-protein complex by lncRNA	*Lnc TCF7*	Liver cancer	Promoting the self-renewal of CSCs and tumor proliferation	Wang Y. et al., 2015
	*Lnc*β*-Catm*	Liver cancer	Promoting the interaction between EZH2 and β-catenin	Zhu et al., 2016
	*Lnc LBCS*	Bladder cancer	Inhibiting the tumor initiation and CSCs self-renewal ability	Chen et al., 2019
Induction by endothelial cells	*Bmi-1*	Head and neck squamous cell carcinoma	Promoting the stemness maintenance of CSCs	Krishnamurthy et al., 2010
Induction by macrophages	*Stat3*	Murine breast cancer	Increasing the expression of the stemness maintenance gene SOX2	Yang et al., 2013
Induction by CAFs	*Fgf4*	Ovarian cancer	Maintaining self-renewal capability of CSCs	Yasuda et al., 2014

### Changes in Stem Cell Signaling Pathways

It is worth mentioning that the Wnt pathway is closely involved in the regulation of stemness in both somatic stem cells and CSCs (Matsui, 2016). A recent study demonstrated that tribbles pseudo-kinase 3 (TRIB3) induces and maintains stemness of colorectal cancer stem cells (CCSCs) by changing the homeostasis of CSCs’ self-renewal and differentiation, which is mediated by forming a positive feedback loop between TRIB3 and Wnt/β-catenin. Upregulation of TRIB3 expression enhances CCSCs properties, including prokaryotic size, continuous passage ability, and tumor initiation frequency (Hua et al., 2019). Consistent with this finding, our recent study also indicates that WNT/β-Catenin signaling can preferentially direct symmetric cell division, one of the major regulatory signaling for a hierarchy of prostate epithelial cells (Wang et al., 2014), in hTERT^high^ PCSCs to improve the self-renewal (Zhang K. et al., 2017). In addition, a more recent study shows that plant homeodomain finger-containing protein 20 (PHF20) is a crucial epigenetic regulator for sustaining the stem cell-like phenotype in GBM via regulation of the degradation of β-catenin in a WNT1 inducible signaling pathway protein 1 (WISP1)/biglycan (BGN) pathway dependent manner (Ma et al., 2020). Also ASCL1 was reported to promote Wnt signaling to induce Wnt target gene AXIN2 by inhibition of Dickkof-1 (DDK1) for the maintenance of GSCs (Rheinbay et al., 2013). Besides Wnt, the SHH pathway is also a key player in the stemness maintenance of CSCs (Samadani and Akhavan-Niaki, 2015). For example, it is reported that treatment of pancreatic cancer stem cells with GANT61, an inhibitor of GLI1 and GLI2, two SHH signaling downstream effectors can effectively reduce the characteristics of cancer stem cells. Furthermore, a simultaneous therapy of mechanistic target of rapamycin kinase (mTOR) inhibitor with GANT61 was confirmed as a more effective treatment for pancreatic cancer by inhibition of the stemness of pancreatic cancer stem cells (Miyazaki et al., 2016).

### DNA Damage Repair

Recently a DNA damage repair related ubiquitin-binding enzyme RAD6 is found to regulate mutagenic DNA damage tolerance to respond to various genomic damages, including chemotherapy and radiation therapy in ovarian cancer. Elevated RAD6 expression promotes the development of stem cell-like phenotypes and resistance to carboplatin by stimulating the monoubiquitination of FA complementation group D2 (FANCD2) and proliferating cell nuclear antigen (PCNA), both of which are important for drug-induced DNA crosslink repair and DNA damage tolerance mechanisms, respectively (Somasagara et al., 2017).

### DNA and Histone Methylation

DNA methylation is also an important epigenetic regulatory mode to regulate the stemness of CSCs. For example, it is revealed that DNA methyltransferase improves the stemness of colorectal CSCs and that reversely its inhibitor 5-Aza-2′-deoxycytidine (5-AzaDC) reduces the abundance of colorectal CSCs by downregulation of DNA hypermethylation of the canonical Wnt pathway (Li et al., 2018). Similar to DNA methylation, histone methylation modifying enzymes can promote gene expression to improve the stemness of CSCs (Norollahi et al., 2019). It has been reported that protein arginine methyltransferase 5 (PRMT5), an arginine methyltransferase, can be recruited to the promoter of forkhead box P1 (FOXP1) to facilitate H3R2me2s, SET1 recruitment, H3K4me3, and FOXP1 expression in BCSCs so that the number of BCSCs is increased (Chiang et al., 2017). Another famous histone lysine methyltransferase is enhancer of zeste 2 polycomb repressive complex 2 (Ezh2), which is responsible for catalyzing methylation of lysine 27 (H3K27) of histone H3 and is indispensable for the maintenance and proliferation of cancer stem cells (Wen et al., 2017). Studies on both GSCs and BCSCs have found that knock-down of Ezh2 or inhibition its methyltransferase activity can inhibit the self-renewal and tumorigenetic ability of GSCs (Suva et al., 2009; van Vlerken et al., 2013). Mechanistically, Ezh2 can inhibit the ability of DNA break repair by reducing expression of DNA break repair genes to promote the growth of CSCs (Stefansson and Esteller, 2011).

### MicroRNA

Accumulating evidences reveal that microRNAs play key roles in regulation of not only CSCs’ initiation but also the maintenance of CSCs’ stemness (Fang et al., 2015). For instance, ectopic expression of miR-30 can inhibit the self-renewal ability of BCSCs in breast cancer xenografts to reduce tumor occurrence and metastasis (Yu et al., 2010). On the other hand, as Drosha is essential for the biosynthesis of microRNA, abnormal expression (Zhang et al., 2016) or function-loss of Drosha (Xu et al., 2017) are related to the malignancy of tumors. In our previous studies, we found that in prostate cancer impaired recruitment of Drosha to the precursor of miR-7 (pri-miR-7) reduces production of mature miR-7, which leads to overexpression of its target gene Kruppel like factor 4 (KLF4), one of the key stemness gene, to enhance the stemness of PCSCs for promotion of tumor growth and metastasis (Chang et al., 2015). However, a recent study has reported an unconventional microRNA, miR-6778-5p, which functions in a Drosha independent matter and acts as an important regulator for maintaining CSCs stemness in Drosha-silenced or low expressed gastric cancer (GCa). It has been found that in Drosha silenced GCa cells, miR-6778-5p positively regulates expression of its host gene serine hydroxymethyltransferase 1 (SHMT1), a key regulator in the folate-dependent serine/glycine interconversion, to mediate the compensatory activation of cytoplasmic carbon metabolism and in turn plays an important role in maintenance of the stemness of gastric cancer stem cells (GCSCs) (Zhao et al., 2020).

### LncRNA

Over past decades, increased studies have revealed that lncRNAs act as one of the key regulators to maintain the stemness of CSCs via formation of multiple functional RNA-protein complex (Castro-Oropeza et al., 2018). In HCC, LncTCF7 is upregulated in liver cancer stem cells (LCSCs) for regulation of LCSCs self-renewal and tumor proliferation. Mechanistically, LncTCF7 recruits the SWI/SNF complex to anchor on the promoter of its host gene TCF7 and improves the tumorigenic activity of LCSCs by activation of TCF7 transcription and Wnt signaling (Wang Y. et al., 2015). Studies have also shown that lnc β-Catm can promote the interaction between EZH2 and β-catenin and enhance the methylation of β-catenin by EZH2. Methylation of β-catenin inhibits its phosphorylation and ubiquitination, and eventually activates Wnt/β-catenin pathway to enhance the stemness of LCSCs (Zhu et al., 2016). Unlike oncogenic lncRNAs, tumor suppressive lncRNAs are usually repressed to attenuate their inhibitory effect on the stemness of CSCs. For example, Lnc-LBCS is observed to be significantly downregulated in bladder cancer stem cells, which inhibits the tumor initiation and CSCs self-renewal ability *in vivo* and *in vitro*. Lnc-LBCS directly binds to hnRNPK and EZH2 to form a complex, by which mediates H3K27me3 to inhibit the transcription of SOX2, an essential transcriptional factor for the self-renewal of CSCs (Chen et al., 2019).

### Endothelial Cells

Increasing evidences show that the factors secreted by endothelial cells play an important role in the self-renewal and survival of CSCs (Krishnamurthy et al., 2010). It is reported that Bmi-1 secreted by endothelial cells can promote the stemness maintenance of ALDH^+^CD44^+^ stem-like cells in HNSCC (Krishnamurthy et al., 2010). Also endothelial cells was found to secrete vascular secretion factors and activate the NOTCH pathway in long-term hematopoietic stem cells (LT-HSCs) through a direct cell-to-cell contact, thereby stimulating the growth of LT-HSCs and contributing to their stemness maintenance (Butler et al., 2010).

### Macrophages

Previous studies have proven that macrophages can be recruited into tumor tissues and be transformed into tumor-associated macrophages (TAMs), thereby providing a favorable microenvironment for the occurrence and development of cancer (Hashimoto et al., 2016). These TAMs not only prevents T cells from attacking tumor cells, but also secretes a series of inflammatory factors to enhance the stemness of tumor cells (Chen et al., 2018). For example, for the purpose of enhancing the expansion and tumorigenic potential of CSCs, TAMs secrete IL-6 and EGF to activate the STAT3 signaling in CSCs to enhance expression of the stemness gene SOX2 (Yang et al., 2013).

### CAFs

It has been reported that co-inoculation of fibroblasts with ovarian cancer stem cells (OCSCs) in nude mouse significantly increases the tumorigenesis ability *in vivo* caused by an elevated expression of fibroblast growth factor 4 (FGF4) to maintain self-renewal capability in OCSCs (Yasuda et al., 2014). Similarly, CAFs can also promote expression of CSCs markers through TGF-β signaling to maintain the stemness of scirrhous gastric cancer cells (Hasegawa et al., 2014). In addition, CAFs derived from lung cancer patients are found to have potential to maintain the stemness of LCSCs in a paracrine manner through the insulin-like growth factor II (IGF-II)/IGF1 receptor (IGF1R)/Nanog axis (Chen et al., 2014).

## Regulation of Therapeutic Resistance of CSC

**FIGURE 3:**
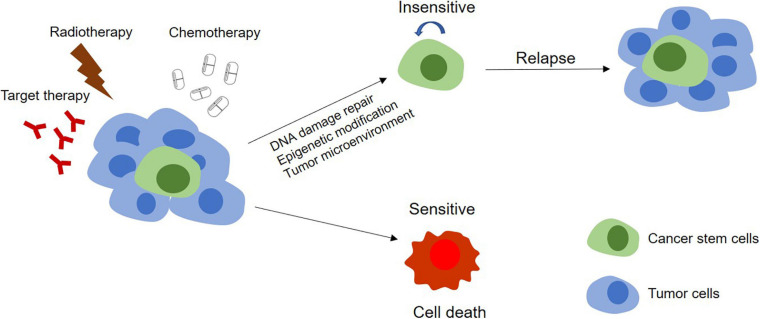
Models of tumor therapeutic resistance. In CSCs model, therapy resistance can be mediated by stem cells. In this model, the tumor contains a small population of CSCs and their differentiated offsprings. With chemotherapy, radiotherapy, or targeted therapy, the DNA damage repair, epigenetic modification, and the effect of the tumor microenvironment on these CSCs make themselves become insensitive to treatments and survive the therapy. The completely differentiated tumor cells are sensitive to treatments and will be killed. Surviving CSCs can further proliferate and differentiate, leading to tumor relapse.

A major challenge of cancer therapy is the resistance to chemotherapy, radiotherapy, and anti-tumor drugs, which is mainly caused by the existence of CSCs (Figure 3). CSCs can keep themselves at a dedifferentiated state so to resistant to the treatment by overexpression of therapeutic resistance related genes and improvement of the ability of DNA damage repair, by deregulation of related non-coding RNAs and by interaction with other types of adjacent cells in microenvironment as well (Table 3).

**TABLE 3:** Genes involved in the therapy resistance of CSCs.

**Regulation model**	**Gene**	**Cancer type**	**Function**	**References**
Gene mutation	*Cd133*	Lung cancer Glioblastoma Prostate cancer	Regulating CSCs through Notch pathway	Liu et al., 2013
	*Aldh1*	Non-small cell Lung cancer	Driving CSCs into a dormant state	Dai et al., 2016
	*Pi3k*	Cervical cancer	Driving CSCs out of a dormant state	Jo et al., 2008
	*Cd10*	Head and neck cancer	Inducing overexpression of OCT3/4	Fukusumi et al., 2014
	*Cd271*	Esophageal squamous cell carcinoma	Resistanting to DDP and 5-FU treatment in CSCs	Li et al., 2015
Regulation by microRNA	*miR-450B-5P*	Colorectal cancer	Inhibiting the resistance to chemotherapy	Jin et al., 2016
Gene transcription changed by lncRNA	*NRAD1*	Triple-negative breast cancer	Promoting tumor growth and resistance to chemotherapy	Vidovic et al., 2020
	*H19*	Colorectal cancer	Activating β-catenin and inducing inhibition the stemness of CSCs	Ren et al., 2018
	*Stat3*	Liver cancer	Enhancing the expression of stemness associated genes and the potential of chemotherapy resistance	Wan et al., 2014
Induction by CAFs	*Cd44*	Several cancers	Relating to the antioxidant effect of CSCs	Kinugasa et al., 2014

### Abnormal Expression of Therapeutic Resistance Related Genes

Previous studies have shown that high expression of CD133 is not only one of the most common markers for CSCs, but also tightly related to their capability of self-renewal as well as drug-resistance (O’Brien et al., 2007). Analysis of lung cancer xenograft tissue showed that cisplatin treatment increases the proportion of CD133^+^ cells caused by increasing the cleavage of Notch1 to activate the Notch pathway. Treatment with γ-secretase inhibitor DAPT or Notch1 specific shRNA can significantly inhibit the enrichment of cisplatin-induced CD133^+^ cells and enhance their sensitivity to doxorubicin and paclitaxel (Liu et al., 2013). Similar to this finding, our recent study also demonstrated that inhibition of the Notch pathway appears to be a promising adjuvant therapy of androgen deprivation therapy (ADT) for prostate cancer (Cui et al., 2018). In addition, it has also been shown that CD133^+^ glioblastoma is more effective in repairing DNA damage than CD133^–^ cells with more sensitive checkpoint activation (Bao et al., 2006). Based on the fact that a dormant state of CSCs plays a key role in resistance of anti-tumor drugs (Kleffel and Schatton, 2013). Dai et al. (2016) found that increased expression of stem cell marker genes such as CD133 and ALDH1 can drive CSCs into the dormant state against the cytotoxicity of 5-fluorouracil (5-FU) in non-small cell lung cancer. On the other hand, inhibition of PI3K-AKT signal also causes dormancy. Under the condition of nutritional deficiency, the cancer cell-derived factors can promote CSCs’ dormancy by inhibition of PI3K to induce cell autophagy (Jo et al., 2008). Besides CD133, overexpression of another CSC marker CD10 was reported to enhance the characteristics of CSC in HNSCC cells and plays an important role in anti-chemotherapy and anti-radiotherapy by inducing overexpression of OCT3/4 (Fukusumi et al., 2014). In addition, elevated expression of CD271, a member of tumor necrosis factor receptor superfamily, in CSCs enables them to survive better by resisting to DDP and 5-FU treatment (Li et al., 2015).

### DNA Damage Repair

It has been reported that expression of multiple reactive oxygen species (ROS) scavengers, such as superoxide reductase and glutathione reductase (Skvortsov et al., 2015; Skvortsova et al., 2015), is upregulated in CSCs to reduce ROS-induced DNA and cell damage (Wang et al., 2013), so that the ROS-mediated apoptosis is prevented and the efficacy of chemotherapy and radiotherapy is attenuated (Dawood et al., 2014).

### MicroRNA

It has been well-known that enhanced stemness of CSCs by deregulation of microRNA is involved in the treatment resistance in multiple cancers (Mens and Ghanbari, 2018). For example, it has been reported that expression of miR-450B-5P is significantly downregulated in recurrent CRC tissues to increase expression of its direct target SOX2 to enhance the stemness of CSCs and the resistance to chemotherapy (Jin et al., 2016). In addition, a recent study has shown that miR-29c-3p downregulates ATG14 by inducing the expression of FOXP1 to inhibit autophagy and promote cisplatin resistance in ovarian cancer cells (Hu et al., 2020).

### LncRNA

It has been found in triple-negative breast cancer that a non-coding RNA, NRAD1, which is regulated by aldehyde dehydrogenase 1A3 (ALDH1A3) and acts as its downstream effector, plays a role in enhancement of CSCs characteristics, promotion of tumor growth and resistance to chemotherapy via binding to the chromatin to change gene transcription (Vidovic et al., 2020).

### Macrophages

Accumulating studies demonstrated that macrophages or TAMs can induce therapeutic resistance in tumor via an interaction with CSCs (Sainz et al., 2016). It has been reported that IL-6 secreted by TAMs induces cell proliferation of CD44^+^ liver CSCs in its microenvironment by activating STAT3 PAGE signaling to enhance the expression of stemness associated genes and the potential of chemotherapy resistance (Wan et al., 2014). Similarly, TAMs can directly induce the stem cell-like properties and chemoresistance of pancreatic duct adenocarcinoma by activating signal transducer and activator of transcription 3 (STAT3) signal. Inhibition of TAMs by M-CSF or chemokine ligand 2 (CCL2) receptor inhibitors can lead to a reduction of the number of CSCs and improve the efficacy of chemotherapy (Mitchem et al., 2013).

### CAFs

It was reported that a CD10^+^GPR77^+^ CAFs subgroup can support tumor cells to promote resistance to chemotherapy by providing CSCs a suitable nest for growth (Su et al., 2018). In addition, it has been found that a large number of CD44^+^ CAFs are usually located in the hypoxic part of the tumor tissue. Data revealed that these CD44^+^ CAFs can produce and secrete several soluble factors which are related to the antioxidant effect of CSCs after they are absorbed by CSCs (Kinugasa et al., 2014). Besides a direct secretion of soluble factors, CAFs can also transfer molecules into CSCs mediated by exosomes, which induces therapeutic resistance of CSCs. For example, it has been reported that CAF delivers LncRNA H19-containing exosomes into CSCs. LncRNA H19 can activate β-catenin and act as an endogenous competitive RNA of miR-141 in CRC against the miR-141 induced inhibition on the stemness of CSCs.

## Conclusion

**FIGURE 4:**
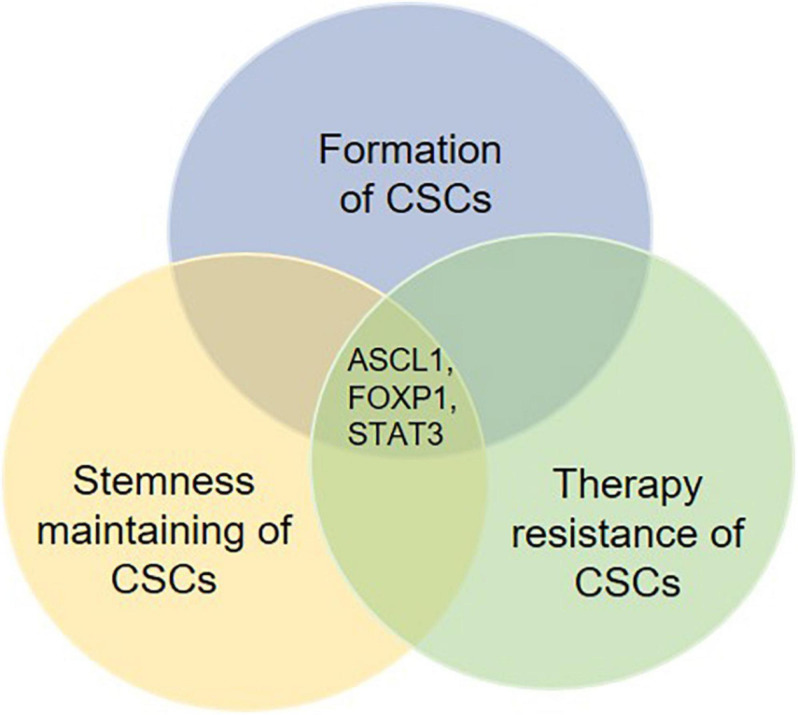
Potential key regulatory factors governing the fate determination of CSCs. Several molecules are found to be involved in not only the formation, the stemness maintenance but also the therapy resistance of CSCs. These molecules such as ASCL1, FOXP1, and STAT3 may work as key regulatory factors for fate determination of CSCs. Therefore, therapies targeting such molecules are likely to show an effective effect post-treatment.

Mounting evidence indicate that the formation and stemness maintenance of CSCs is a complicated process, which is caused by a synergistic regulation of multiple intracellular genetic and epigenetic effectors in cancer in combination with inducers from tumor microenvironment. In addition, these related regulatory mechanisms are directly or indirectly involved in the development of therapeutic resistance. Moreover, several key regulatory factors can regulate not only the formation and self-renewal, but also the therapy resistance of CSCs (Figure 4). Therefore, it would be important for future studies to focus on these key factors. Targeting these key factors may be a good therapeutic strategy for the management of cancer by suppressing formation and self-renewal of CSCs’ and facilitating the differentiation and elimination of CSCs.

## Author Contributions

NJ searched for original articles and wrote the manuscript. NJ, Y-XF, and W-QG provided the idea and designed the review. All authors reviewed the manuscript and approved the final manuscript.

## Conflict of Interest

The authors declare that the research was conducted in the absence of any commercial or financial relationships that could be construed as a potential conflict of interest.

## Abbreviations

ABCG2: ATP binding cassette subfamily G member2
ALDH: aldehyde dehydrogenase
ALDH1A3: aldehyde dehydrogenase 1A3
ALKBH5: alkB homolog 5
AR: androgen receptor
ASCL1: Achaete-Scute Complex-Like 1
ATG14: autophagy related 14
BCSCs: breast cancer stem cells
CCL2: chemokine ligand 2
CCSCs: colorectal cancer stem cells
CDKN1C: cyclin dependent kinase inhibitor 1C
CRC: colorectal cancer
CREBBP: Cyclic AMP response element binding protein
CSCs: cancer stem cells
DDK1: Dickkof-1
DNMT: DNA methyltransferase
E2F1: E2F transcription factor 1
EZH2: enhancer of zeste 2 polycomb repressive complex 2 subunit
FANCD2: FA complementation group D2
FGF4: fibroblast growth factor 4
FOXM1: forkhead box M1
FOXP1: forkhead box P1
GATA3: GATA binding protein 3
GBM: Glioblastoma
GCa: gastric cancer
GCSCs: gastric cancer stem cells
GLI1: GLI family zinc finger 1
GLI2: GLI family zinc finger 2
GPR371L: G protein-coupled receptor 37-like 1
GSCs: glioblastoma stem cells
H3K27me3: histone H3 lysine 27 tri-methylation
HAND2: heart and neural crest derivatives expresses 2
HCC: hepatocellular carcinoma
HGF: hepatocyte growth factor
HMGA2: high mobility group AT-hook 2
hnRNPK: heterogeneous nuclear ribonucleoprotein K
HNSCC: head and neck squamous cell carcinoma
HOXC8: homeobox C8
IFN-γ: interferon-γ
IGF-II: insulin-like growth factor II
IL-33: interleukin 33
IL-6: interleukin 6
JAK2: janus kinase 2
JMJD1C: jumonji domain containing 1C
KDM3A: lysine demethylase 3A
KDM3B: lysine demethylase 3B
KLF4: Kruppel like factor 4
LCSCs: liver cancer stem cells
LPECs: liver parenchymal endothelial cells
LT-HSCs: long-term hematopoietic stem cells
MaSCs: mammary stem cells
M-CSF: macrophage colony stimulating factor
MEG3: maternally expressed 3
MORC2: MORC family CW-type zinc finger 2
mTOR: mechanistic target of rapamycin kinase
MYT1: myelin transcription factor 1
NANOG: nanog homeobox
NANOGP8: Nanog homeobox retrogene P8
NF-kB: nuclear factor kappa B
NKX6-2: NK6 homeobox 2
NSCLC: non-small cell lung cancer
OCSCs: ovarian cancer stem cells
OCT4: octamer-binding transcription factor 4
OCT6: octamer-binding transcription factor 6
PAK1: p21 (RAC1) activated kinase 1
Par3: pulmonary adenoma resistance 3
PCNA: proliferating cell nuclear antigen
PCSCs: prostate cancer stem-like cells
PHF20: plant homeodomain finger-containing protein 20
PHOX2B: paired like homebox 2B
Prlr: prolactin receptor
PRMT5: protein arginine methyltransferase 5
PTK7: protein tyrosine kinase 7
SHMT1: serine hydroxymethyltransferase 1
SKP2: S-phase kinase associated protein2
SOCS3: suppressor of cytokine signaling 3
SOX2: SRY-box transcription factor 2
STAT3: signal transducer and activator of transcription 3
STAT5: signal transducer and activator of transcription 5
SUV39H1: histone H3 lysine 9 methyltransferase
TCF7: transcription factor 7
TRIB3: tribbles pseudokinase 3
WISP1: WNT1 inducible signaling pathway protein 1
Zeb1: zinc finger E-box binding homeobox 1.
